# Supplementary material for: Multimorbidity and health system priorities in Zimbabwe: A participatory ethnographic study
Source: PLOS Glob Public Health. 2025 Apr 28;5(4):e0003643. doi: 10.1371/journal.pgph.0003643 (PMC12036853; doi:10.1371/journal.pgph.0003643)
Supplement: S1 Table — Provides information about the demographics of PLWMM included in the study, including age, sex, province, and conditions. (DOCX) [file pgph.0003643.s001.docx]

**S1 Table.** Breakdown of participant demographics of people living with multimorbidity (PLWMM)

| **Variable** | **Value** | | | | | |
| --- | --- | --- | --- | --- | --- | --- |
| Age range | 19-87 | | | | | |
| Gender – n(%) female | 14(61%) | | | | | |
| HIV status – n(%) HIV+ | 13(56%) | | | | | |
| Province | Harare = 8, Mashonaland East = 5, Bulawayo = 5, Matabeleland South = 5 | | | | | |
| Condition profiles | HIV & diabetes mellitis (DM) | HIV & arthritis | DM, hypertension (HPT) & blind | HIV, prostate enlargement, DM, angina | DM, cardiac failure & systemic lupus | HIV & HPT |
|  | HIV, HPT & asthma | HIV & asthma | HIV, prostate cancer, chronic cardiac failure | HIV, HPT, asthma, chronic ulcers | HPT & DM | HIV, epilepsy & asthma |
|  | HPT & Ascites | HPT & chronic cardiac failure | DM & chronic liver disease | HIV, epilepsy, HPT, asthma |  |  |
