# Supplementary material for: Multimorbidity and health system priorities in Zimbabwe: A participatory ethnographic study
Source: PLOS Glob Public Health. 2025 Apr 28;5(4):e0003643. doi: 10.1371/journal.pgph.0003643 (PMC12036853; doi:10.1371/journal.pgph.0003643)
Supplement: S2 Table — Provides select quantitative findings from the health facility survey regarding medicines, equipment, and training for NCDs. (DOCX) [file pgph.0003643.s002.docx]

**S2 Table. Availability of medicines, equipment, diagnostics, and training for common NCDs in 30 health facilities**

| **Disease** | **Medicines* (available and in-date)** | **Equipment & diagnostics (available & functional)** | **Training (in last two years)** |
| --- | --- | --- | --- |
| Diabetes | Metformin (27/30) , glibenclamide (27/30), regular insulin injection (11/30) | Glucometer (28/30), glucose strips (23/30) | (6/30) |
| Hypertension | Hydrochlorothiazide (29/30) , ACE inhibitor (10/30), beta blocker (6/30), furosemide (15/30) | Blood pressure (BP) machine (30/30)* | (6/30) |
| Chronic respiratory disease | Inhaled bronchodilator (28/30), inhaled steroid (3/30), prednisolone (11/30) | Peak flow meter (5/30), spacers for inhalers (17/30) | (5/30) |
| Depression | First-line antidepressant (23/30), second-line antidepressant (4/30) | - | (10/30) |

* Respondents frequently noted that medicine might be here today, but not tomorrow.

** Insufficient BP machines even if present: “there are inadequate blood pressure machines to accommodate the whole clinic” (Public clinic, Bulawayo)
