# Supplementary material for: Multimorbidity and health system priorities in Zimbabwe: A participatory ethnographic study
Source: PLOS Glob Public Health. 2025 Apr 28;5(4):e0003643. doi: 10.1371/journal.pgph.0003643 (PMC12036853; doi:10.1371/journal.pgph.0003643)
Supplement: S3 Table — Presents the main priorities and focal institutions for responding to multimorbidity identified during the final collaborative workshop held in Harare 1st December 2023. (DOCX) [file pgph.0003643.s003.docx]

**S3 Table. Priorities and key institutions for responding to multimorbidity in Zimbabwe**

Extracted from: Dixon, Justin et al., 2024, "Multimorbidity in Zimbabwe: Evidence and Priorities Dialogue: Workshop Report. 01 December 2023, Harare, Zimbabwe", <https://doi.org/10.7910/DVN/ANDL1N>, Harvard Dataverse, V1

| **Priority area** | **Specific priorities & additional notes** | **Key Institutions**  **/ departments** |
| --- | --- | --- |
| **1.** Pooled/integrated financing | Greater overall investment in health   - In accordance with Abuja Declaration (15% GDP) | Policy, Planning, & Health Economics (CD Public Health)  Partners / funders |
|  | Pooled funding from partners   - Break from the model of funding mechanisms for particular diseases and rather fund the healthcare system as one - May need a staged approach to pooled funding to generate buy-in and minimise resistance |  |
|  | Costing of multimorbidity care   - Costing dyads, e.g. cancer and mental health, diabetes and hypertension, etc. |  |
| **2.** Integrated, bottom-up governance | Reinvigoration of the primary healthcare emphasis   - Rebalancing with current prioritisation of quaternary services | Policy, Planning, & Health Economics  (CD Public Health) |
|  | Policy to precede financing rather than vice versa   - Disease-specific funding creates misalignment between policy and needs/disease burden - Moving away from enclaving of disease-specific funding within units/programmes |  |
|  | National integration framework for multimorbidity   - Potentially starting small-scale at sub-national level and building towards a national framework. |  |
|  | Dynamic, bottom-up policymaking continuously learning from ground-level experience   - Strengthen alignment of policymaking and local realities of care seeking/delivery, greater inclusivity and feedback loops - Stakeholder engagement needed on benefits of service integration |  |
|  | Multisectoral collaboration and strategy   - Particular emphasis on multimorbidity prevention initiatives - Engagement with traditional medicine |  |
| **3.** Holistic, decentralised prevention and care for multimorbidity | Improved awareness, information through health promotion   - Currently little awareness of NCDs and multimorbidity at community level as for HIV - Opportunities to address shared risk factors for multiple conditions through multisectoral efforts | Health Promotion (CD Public Health) |
|  | Integrated screening at community and facility level   - Emphasis on vulnerable and high-risk groups (e.g. elderly, socio-economically marginalised, stigmatised groups) - Recognition that screening is not effective without linkage to and availability of care | Directorates under CD Public Health (NCDs, HIV&TB, Mental Health, etc.)  CD Curative Services |
|  | Chronic/multimorbidity clinics at primary level   - Challenges created with separate care for HIV and through OI clinics - Need for ‘one stop shop’ chronic care models for all chronic conditions/multimorbidity - Opportunity to leverage OI chronic care infrastructure by expanding inclusion to all chronic/multimorbidity patients - Leveraging the South African experience with Integrated Chronic Disease Management (ICDM)(44) |  |
|  | Ensuring medicines, commodities, equipment, and other tools for multimorbidity management   - All presentations highlighted severe resource shortage as impediment to multimorbidity prevention, diagnosis, and care |  |
|  | Removal of user fees for chronic / vulnerable patients   - User fees at clinics and hospitals undermines continuity of chronic care - Revival of social safety net for waiving of user fees |  |
|  | Capacitation of staff in NCDs, mental health, and current HIV-NCD integration guidance | CD Human Resources  Health Service Commission  Medical training Institutions |
|  | Retention of staff /skills   - Incentivising trained staff to stay in public sector through funding, rewards, and continuous engagement - Integrated training for all staff lessens consequences when individuals move on |  |
|  | Greater emphasis and valuation of generalist skillsets   - Current aspirations among medical trainees towards (super-)specialism - Need for valuation and motivation of generalist cadres working at lower levels of care |  |
|  | Skills shifting and multidisciplinary teams   - Nurse/community initiation of treatment for certain NCDs, as for ART - Non-hierarchical relationship between cadres/levels of care to foster team building - Caution with overburdening lower-level cadres |  |
| **4.** Integrated, person-centred health data and research | Improve NCD estimates   - Currently NCD data lagging behind HIV and partner-supported health conditions | Health Informatics  National Institute for Health Research  Academic Institutions |
|  | Improve visibility of multimorbidity within routine health information and M&E systems at all levels   - Strengthened and harmonized M&E platform across programmes - Expansion of reporting from cross-sectional single disease counts to person-centred and multimorbidity data across the life course - Utilisation of Electronic Health Records (EHR) to improve both user experiences and multimorbidity data capture/reporting - Inclusion of and feedback loops with ground-level perspectives and experiences on multimorbidity care |  |
|  | Strengthening integration and synergies between academic institutions and MoHCC   - Bidirectional effort – strengthened researcher engagement/dissemination (academia) and conveyance of research needs and uptake of findings (MoHCC) - Elevation of applied health systems research with policy focus |  |
|  | Broader value shift in the kinds of knowledge that counts in decision-making/policy   - Deliberative and experiential knowledge as well as information/data - Practical learning and confidence to experiment with new ideas supports continuous learning and self-reliance |  |
