# Supplementary material for: Multimorbidity and health system priorities in Zimbabwe: A participatory ethnographic study
Source: PLOS Glob Public Health. 2025 Apr 28;5(4):e0003643. doi: 10.1371/journal.pgph.0003643 (PMC12036853; doi:10.1371/journal.pgph.0003643)
Supplement: Attachment — Submitted filename: PLOS-GPH_Response_Letter_15_01_24.docx [file pgph.0003643.s006.docx]

Dear Editors of PLOS Global Public Heath,

**Re: Assembling the Challenge of Multimorbidity in Zimbabwe: A Participatory Ethnographic Study**

We would like to express our gratitude for the insightful and constructive reviewers’ and additional editors’ comments on our paper, “Assembling the Challenge of Multimorbidity in Zimbabwe: A Participatory Ethnographic Study”. We have revised our manuscript accordingly and believe that the paper is much strengthened as a result. We hereby resubmit with highlights to mark alterations. Following the reviewer and editor’s comments, the main changes have been made to expand the methodology and to improve the clarity and accessibility of the language, with other revisions throughout. In addition to the reviewers’ and editors’ comments, we have made minor edits to ensure that the document is consistent with the changes made. Finally, we have revised the figures to ensure that they are high quality and that they meet the journal’s open access policies. Below, we respond to each of the reviewer’s and editor’s points in turn.

We hope that you will agree that the paper is now strengthened and ready for publication.

Yours faithfully,

Justin Dixon, on behalf of the authors

| **Reviewer comment** | **Authors’ response** |
| --- | --- |
| **Reviewer 1** |  |
| **1.** This ethnographic study including the perspectives of the main actors involved (from policymakers to health professionals and patients) shows a potential useful framework to design a more person-oriented health care system in Zimbabwe.  There is a small mistake in page 48, legend should be Figure 2. | We thank the reviewer for their favourable view of the paper and for pointing out this error we have revised the figure legend to read ‘figure 2’. We also found a similar error on line 586 and have corrected the figure number from figure 4 to figure 3. |
| **Reviewer 2** |  |
| **2.** The title does not convey the purpose of this study. In particular, I do not understand the phrasing “Assembling the Challenge” of Multimorbidity. Maybe this is a phrase that is meaningful in the social sciences field at large – but that phrase has no meaning to me in medical science.  Could the authors choose a title that has more “stand-alone” meaning to readers?  For example, to be parallel with the authors' stated intent: “Multimorbidity and healthcare priorities in Zimbabwe: A participatory ethnographic study” | Thank you for the comment. We agree that the title assumes too much in advance of reading the article, and we have therefore taken the reviewer’s suggestion to rename. We have used the title: “Multimorbidity and health system priorities in Zimbabwe: A participatory ethnographic study”. We have chosen to phrase as ‘health system’ rather than ‘healthcare’ priorities since we examine the broader health system beyond care delivery. Thank you for the valuable suggestion. |
| **3.** Much of the English is written in a circuitous and non-linear fashion. For example, phrases offset with commas could be minimized and written in a style that does not break the reading flow. As currently written, much of the Introduction and Methods come across as disconnected ideas that could be better integrated into a clear and “tight” summary.  For example:  [ABSTRACT, lines 59-60] “Multimorbidity, we found, was new to many…” can simply be written as “We found that multimorbidity was new to many…”  [INTRODUCTION, lines 102-103] “Bosire et al, for instance, demonstrated…” can be simply written as “For instance, Bosire et al. demonstrated…”  There are numerous examples of this nested phrase style throughout the article that would benefit from heavy handed editing. | Thank you for pointing out that the English is often circuitous and non-linear. We have gone through the manuscript in detail and revised all such sentences, with particular attention to the introduction and methods. As suggested, our changes include (but go beyond) the following:  “Multimorbidity, we found, was new to many…” has been changed to “We found that multimorbidity was new to many…” (Abstract, line 59)  “Bosire et al, for instance, demonstrated…” has been changed to “For instance, Bosire et al. demonstrated…” (line 113) |
| **4.** I commend the authors for thinking about the complex and non-trivial aspects of multimorbidity as a construct in guiding (or prioritizing) medical practice. This is a unique endeavour and has potential to offer a model for other regions.  While I find the article to be interesting, I also find there to be very little quantitative or even qualitative data that can be scientifically and rigorously summarized. I am not a qualitative or social science expert, so my ability to remark on these findings is limited. The English is at times difficult to follow and circuitous. I think the paper could benefit from more robust organization. I encourage the authors to remove extraneous terms that are not relevant to their specific hypotheses or goals. The paper could probably be half of its current length with better organization and substantial editing and focus. | Thank you for the favourable view on our article and its approach to the evaluating the utility of multimorbidity construct for guiding medical practice in Zimbabwe. We certainly hope that this has potential to shape priority setting in other countries and regions too.  We appreciate the reviewer’s concerns about an apparent lack of data that can be easily summarised. We would clarify that, as an ethnographic study, the limited quantitative data collected is not the focal point for this article (see also response to **comment 10** re. survey data). The narrative style of the article is characteristic of ethnography, which weaves interview, observational, and other data into a ‘thick’ description that, in this case, offers different “windows” into multimorbidity (as we put it line 315) across the health system, combining rich descriptions of social context, the voices of respondents, and our own positioning as researchers. This approach enabled us to characterise the ‘whole-system’ challenge of multimorbidity in a way that would have been challenging (and less compelling) in summary fashion or through other writing styles. We appreciate this may differ from a biomedical or public health approach but is consistent with the research questions we posed and the methodology we have adopted to answer them.  The length is within the limits of what would normally be the case for ethnographies. However, thank you for pointing out the long and circuitous sentences, and other opportunities for making our article more accessible to a multidisciplinary audience. While the overall structure of the results has been retained to remain faithful our data, we have done a tight edit to use more accessible grammar/language and to significantly reduce the word count from **8,540 to 7,781** words (factoring in additions requested in other comments) which we are confident makes the article considerably stronger and more accessible. |
|  |  |
| **Further reviewer comments sent separately** |  |
| **5.** P209 Given the study design, it is important to note the role of participants in the design of the study. For example were facility managers involved in the design of the assessments of health facilities? Had they bought-in to the reasons for doing this assessment? | Thank you for the comments. The study design was based on formative work mapping the emerging field of global multimorbidity research, as well as engagement with the Ministry of Health in Zimbabwe (several of whom are co-authors, as we describe in the methods section). We have clarified this in the opening lines of the methods section (lines 193-196):  *“The design was informed by formative work to map the emerging field of global multimorbidity research[6,17] and engagement in Zimbabwe with the Ministry of Health and Child Care (MoHCC).”*  We did not directly consult facility managers prior to the study, partly because this was beyond the scope of our engagement activities and, in any case, the study was designed precisely to find out whether multimorbidity was of concern to them – a construct that they would ‘buy in’ to. Folding engagement into the study design itself was appropriate given the open-ended, qualitative nature of the study which enabled the continuous iteration of our lines of inquiry in response to participants’ perceptions, experiences. We have clarified how, in lieu of input from facility managers, how the survey was designed lines 214-217:  *“Quantitative and qualitative survey questions were developed based on the WHO Service and Readiness Assessment (SARA) survey,[32] knowledge within our team on NCDs and multimorbidity, and consultation with relevant MoHCC Directorates”.* |
| **6.** It is a little unclear how the focus of the NCDs were selected – four are mentioned – for the health facility assessment. This should be clarified." | Thank you, we have clarified that these the NCDs included in the facility survey were selected because of their inclusion within the WHO’s Service Readiness and Assessment (SARA) survey, to which we also added depression in order to ensure we included a mental health condition. The additions are lines 218-221:  *“…and preparedness for managing specific NCDs (hypertension, diabetes, chronic respiratory disease, and depression.* *These diseases were selected as they are the NCDs included in the SARA survey[32] with the exception of depression, which we added to cover mental health.”.* |
|  |  |
|  |  |
| **Editorial comments** |  |
| **7.** Some figures on prevalence of NCDs and HIV/AIDS in the study setting would be helpful | Thank you, we have included figures on the prevalence of NCDs and HIV in the study setting section. The added sentences on lines 166-169 now read:  *“…Since then, Zimbabwe experienced among the steepest rises in NCDs in sub-Saharan Africa,[27] with an estimated 40% and 13% adult prevalence of hypertension and diabetes, respectively.[28] Modelling also suggests that multimorbidity, particularly among the 12.9% of people in living with HIV, will rise sharply by 2035.[29]”* |
| **8.** PHC facilities are overrepresented in the Facility surveys. Please explain reason for this. In addition, are the selected facilities are public facilities or include private ones also. | Thank you, we can first clarify that all sampled healthcare facilities were in the public and non-for-profit sectors, which we have now clarified lines 212-214:  *“For logistical reasons, all facilities were in the public and not-for-profit sectors, with perspectives from the private sector primarily fed in through other methods”.*  We included more primary care facilities than hospitals because, within our study settings, there are around 10 primary care facilities for every referral hospital (be that secondary, tertiary, or quaternary), and so for each hospital we included a number of the primary facilities from which patients were referred. We might note that there are no secondary or tertiary public sector hospitals in metropolitan provinces, only quaternary ones, hence the referral centres in metropolitan provinces are quaternary hospitals, and those in rural provinces either secondary or tertiary (with referral to quaternary urban hospitals where needed). This is explained narratively in the study setting section, lines 176-183:  *“They [referring to Harare and Bulawayo] also both share a similar provincial health system structure, with primary healthcare for the majority provided by City Councils and most referrals going straight to one of the Central hospitals … Mashonaland East, bordering Harare, is a predominantly rural province in which most public healthcare is run by the central government, which includes primary, secondary, and tertiary care, with referrals for quaternary care generally sent to Harare”.*  As well as including some district hospitals in the selected provinces, we have included all tertiary hospitals within the selected provinces and all bar one quaternary hospitals in the country. Thus, we feel that we have adequately represented the hospital sector within our study sample, within the modest resources available to this study. |
| **9.** Is only the research team involved in the developing facility survey tool or developed in consultation with some participants groups? why only "hypertension, diabetes, chronic respiratory disease, and depression" included as NCD co-morbidity? | Please see response to **comment 5** where we have clarified the sources that fed into the survey design and the reason for the selection of disease conditions. |
| **10.** Why the results from the survey are not presented in the manuscript? How these results complement the qualitative findings? | The survey results were primarily to provide formative knowledge that was then followed up in more detail using qualitative methods. It was also used to start a relationship with healthcare facilities and to generate interest in the follow-up fieldwork activities. We have added a clause to clarify this on lines 209-212:  *“Data collection commenced with a survey of health facilities (n=30) in the four provinces, purposively selected to provide an initial understanding of the capacity of facilities at different levels of care for addressing NCDs and multimorbidity (see Table 2) and to generate interest for follow-up fieldwork”.*  As such, the survey was just as much part of a process of developing relations as it was about the results. We have also clarified in the text that the questions asked during the survey included qualitative questions, which have informed our narrative throughout (lines 214-217):  *“Quantitative and qualitative survey questions were developed based on the WHO Service and Readiness Assessment (SARA) survey,[32] knowledge within our team on NCDs and multimorbidity, and consultation with relevant MoHCC directorates”.*  We might note that surveys were the source of some of quotes presented (e.g. the quote lines 357-360 and the quote lines 560-564). Since we are aware the readership may be interested in a summary of some key quantitative data, it was for this reason that we included summary table S2 presenting the main quantitative survey findings, which we do refer to in the text (e.g. lines 367 and 532) to support the qualitative findings. |
| **11.** More explanation needed on how themes for the interview guides were developed? any framework or prior studies used to guide developing the themes? | Thank you, we have added further detail on the way in which themes for the interview guides were developed, which includes prior qualitative studies on multimorbidity, our knowledge of the context, and ongoing findings after the start of data collection (lines 232-235):  *“Interviews were conducted using bespoke interview guides developed from a set of high-level themes derived from qualitative literature and our knowledge of the context. The guides were adapted throughout the study based on emerging findings.”.*  We have also clarified this in the data analysis section, where explain that data analysis was ongoing through data collection with findings fed back into topic guides (lines 280-282):  *“Analysis was performed on an ongoing, iterative basis by JD and FM that fed back into the ongoing collection of data including through the refinement of interview guides.”* |
| **12.** Revised figures including map. | We have revised the figures, including the map, to ensure that they are high quality and that they meet the journal’s open access policies. In addition to adding the link to the base layer of the map in the legend of figure 1 (see: <https://data.humdata.org/dataset/cod-ab-zwe>), we are happy to also provide the link to the terms of use / license information for the base layer image: <https://data.humdata.org/faqs/licenses> |
